# The Role of Lipid Metabolism Disorders in the Development of Thyroid Cancer

**DOI:** 10.3390/ijms25137129

**Published:** 2024-06-28

**Authors:** Martyna Lukasiewicz, Agata Zwara, Jacek Kowalski, Adriana Mika, Andrzej Hellmann

**Affiliations:** 1 Department of General, Endocrine and Transplant Surgery, Faculty of Medicine, Medical University of Gdansk, 80-211 Gdansk, Poland; martyna.lukasiewicz@gumed.edu.pl (M.L.); hellmannandrzej@gumed.edu.pl (A.H.); 2 Department of Environmental Analytics, Faculty of Chemistry, University of Gdansk, 80-309 Gdansk, Poland; agata.zwara@phdstud.ug.edu.pl; 3 Department of Pathomorphology, Faculty of Medicine, Medical University of Gdansk, 80-211 Gdansk, Poland; jacek.kowalski@gumed.edu.pl; 4 International Centre for Cancer Vaccine Science, University of Gdansk, 80-309 Gdansk, Poland; 5 Department of Pharmaceutical Biochemistry, Medical University of Gdansk, 80-211 Gdansk, Poland

**Keywords:** thyroid cancer, lipid metabolism, fatty acids uptake, fatty acid oxidation, obesity, hormones

## Abstract

Thyroid cancer (TC) is a neoplasm with an increasing incidence worldwide. Its etiology is complex and based on a multi-layered interplay of factors. Among these, disorders of lipid metabolism have emerged as an important area of investigation. Cancer cells are metabolically reprogrammed to promote their rapid growth, proliferation, and survival. This reprogramming is associated with significant changes at the level of lipids, mainly fatty acids (FA), as they play a critical role in maintaining cell structure, facilitating signaling pathways, and providing energy. These lipid-related changes help cancer cells meet the increased demands of continued growth and division while adapting to the tumor microenvironment. In this review, we examine lipid metabolism at different stages, including synthesis, transport, and oxidation, in the context of TC and the effects of obesity and hormones on TC development. Recent scientific efforts have revealed disturbances in lipid homeostasis that are specific to thyroid cancer, opening up potential avenues for early detection and targeted therapeutic interventions. Understanding the intricate metabolic pathways involved in FA metabolism may provide insights into potential interventions to prevent cancer progression and mitigate its effects on surrounding tissues.

## 1. Introduction

Thyroid cancer (TC) is a malignant neoplasm that originates in the thyroid gland and has attracted increasing attention in recent years due to its rising incidence worldwide. The etiology of TC is complex, and a variety of factors contribute to its development. Among these factors, disorders of lipid metabolism have emerged as a notable area of interest. Lipids, including fatty acids (FAs), play a fundamental role in cell structure and function. Consequently, disturbances in lipid metabolism can have profound effects on various cellular processes, potentially favoring the development and progression of TC. Understanding the intricate relationship between lipid metabolism disorders and TC is of paramount importance as it may provide new insights into the prevention and treatment of this malignancy. This review aims to elucidate the intricate interplay between disorders of lipid metabolism and TC and to highlight the possible mechanisms by which lipid disorders contribute to tumorigenesis and their implications for clinical management and therapeutic strategies.

### 1.1. The Thyroid Gland

**Figure 1:**
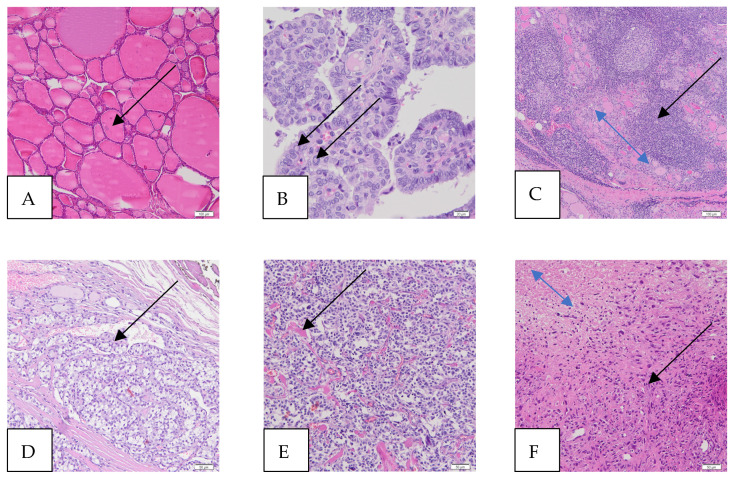
Microscopic images of thyroid and thyroid cancers (hematoxylin and eosin staining—HE). (**A**) Normal thyroid, magnification 100×, scale bar 100 μm (colloid in a follicle, arrow). The thyroid gland consists of many follicles lined by many thyrocytes (follicular cells) and the colloid in the center. The parafollicular cells, called C cells, are sparse and scattered and lie between the follicles (not shown). (**B**) Papillary thyroid carcinoma (PTC), magnification 400×, scale bar 20 μm (intranuclear pseudoinclusions, arrows). The papillae are lined by follicular cells with changes in size and shape, irregularities of the nuclear membrane (intranuclear grooves and intranuclear pseudoinclusions), and chromatin characteristics. The classic (conventional) subtype of PTC (foto) is the most histologic subtype of this cancer. (**C**) Hashimoto’s thyroiditis (HT), magnification 100×, scale bar 100 μm (atrophic follicles, double-headed blue arrow; lymphocytic infiltration, black arrow). HT affects the thyroid gland, causing an increased number of lymphocytes that infiltrate the organ and form primary and secondary lymphoid follicles, which lead to the destruction of thyroid follicles and, thus, a reduction in the amount of colloid, which ultimately causes a deficiency of thyroid hormones. (**D**) Follicular thyroid carcinoma (FTC), magnification 200×, scale bar 50 μm (vascular invasion—the intravascular polypoid tumor is covered by endothelium, arrow). (**E**) Medullary thyroid carcinoma (MTC), magnification 200×, scale bar 50 μm (amyloid deposits in the stroma, arrow). MTC is a malignant tumor derived from the parafollicular C cells of the thyroid gland. (**F**) Anaplastic thyroid carcinoma (ATC), magnification, 200×, scale bar 50 μm (cancer cells, black arrow; cancer necrotic tissue, double-headed arrow). ATC is a highly aggressive thyroid tumor that contains undifferentiated cells and may have focal features of thyroid follicular differentiation. Many pleomorphic cells are spindle, and some have epithelioid shapes.

Histologically, a functional thyroid gland has two main cell types in the parenchyma. The first, the follicular cells, line the colloidal follicles and contribute to iodine concentration and the production of thyroid hormones, iodothyronines (Figure 1A). This cell line contributes to well-differentiated cancers, such as the papillary and follicular types. The second cell line is the C or parafollicular, which is responsible for the production of calcitonin and leads to medullary thyroid cancer (MTC) [1]. Thyroid-stimulating hormone (TSH) released from the anterior pituitary gland stimulates the receptors on the follicular epithelium of the thyroid gland [2].

### 1.2. Thyroid Cancers

The thyroid gland is susceptible to various diseases due to its central role in regulating numerous processes in the body. Autoimmune mechanisms, infections, hormonal imbalances, and genetic predispositions can affect thyroid function and lead to various diseases, including malignancies [3].

Most TCs are diagnosed through ultrasonography followed by a fine needle aspiration (FNA) biopsy of the tumor [4]. The result of the FNA is classified according to the VI grade Bethesda system (Table 1) [5]. The most used staging system for TC is the TNM system of the Union for International Cancer Control (UICC), which is based on three key pieces of information [6].

#### 1.2.1. Papillary Thyroid Cancer (PTC)

PTC is the most common form of TC (80% of all TCs) and can occur at any age. PTC shows an indolent course with a survival rate of 95% over 10 years [6], as most patients respond positively to surgery and targeted therapy [6]. Death from PTC is rare compared to other forms of TC [7]. The clinical presentation of PTC nodules is usually incidental or on routine examination. PTC with a size of ≤10 mm is referred to as PTC microcarcinoma (also known as occult sclerosing carcinoma, occult papillary carcinoma, or incidental PTC) [8]. Patients rarely present with hoarseness or involvement of the cervical lymph nodes. The primary presentation of distant metastases is an unusual finding. The diagnosis should be made based on nuclear morphology rather than architecture [9]. The classic (conventional) subtype of PTC is the most common histologic subtype of this cancer (Figure 1B).

##### Impact of HT on PTC

One of the risk factors for PTC is the chronic inflammation associated with Hashimoto’s thyroiditis (HT) [10], and the coexistence of HT and PTC has been reported with a frequency of up to 62% [11]. Although numerous cellular and clinical studies have confirmed this association, the exact nature of the relationship between these two diseases remains unclear [12]. HT, also known as Hashimoto’s disease, is in fact the most common autoimmune thyroid disorder. In this disease, the immune system produces antibodies against thyroid peroxidase (TPO) and thyroglobulin (Tg). This leads to inflammation and the destruction of the thyroid tissue (Figure 1C) and thus to an underactive thyroid (hypothyroidism), which occurs in 20–30% of patients.

#### 1.2.2. Follicular Thyroid Cancer (FTC)

FTC is the second most common form of TC and accounts for 5–10% of primary thyroid malignancies in non-endemic goitered areas of the world [7]. FTC has been shown to be associated with female sex (M/F = 1:3) and found in areas with iodine deficiency in the diet. The neoplasm is more aggressive than PTC, with a higher risk of metastasis and vascular invasion (Figure 1D).

#### 1.2.3. Medullary Thyroid Cancer (MTC)

MTC is a neuroendocrine neoplasm originating from parafollicular cells (C cells) that produce calcitonin. MTC shows an organoid architecture with a nested growth pattern (Figure 1E). Amyloid deposits are found in the stroma [13]. It accounts for 5% of primary thyroid malignancies. Sporadic or non-familial MTC accounts for 60–70% of cases. The remaining 30% are associated with certain familial syndromes, such as multiple endocrine neoplasia 2A and MEN 2B [14].

#### 1.2.4. Anaplastic Thyroid Cancer (ATC)

Anaplastic thyroid carcinoma (ATC) is an undifferentiated tumor of the follicular epithelium of the thyroid gland. Patients affected by this malignancy are older (mean age 65 years) than patients affected by other types of TC [15]. ATC associated with well-differentiated carcinomas suggests that they originate from the dedifferentiation of well-differentiated TC [7]. ATC is a highly aggressive thyroid tumor that contains undifferentiated cells and may show focal features of follicular differentiation of the thyroid gland (Figure 1F) [16].

### 1.3. Worldwide Burden of TC

TC is the ninth most common malignancy worldwide and the most common endocrine cancer. The etiology of TC is complex, with potential risk factors including female sex, age 40–70 years, family history of thyroid disease, exposure to ionizing radiation, overweight or obesity, hormonal imbalance, and iodine intake. The overall prognosis for TC is very good, as most cases are PTC [17]. According to De Villalonga et al., the median overall TC incidence per 100,000 person-years was 10.2 (6.3–15.0), 15.4 (10.4–21.4) for women and 5.0 (3.5–6.8) for men. The incidences for the histotypes (overall, women, and men, respectively) were 6.0 (2.4–11.3), 11.7 (6.0–19.3), and 4.2 (2.4–6.4) for papillary; 1.1 (0.9–1.4), 1.8 (1.7–1.9), and 0.8 (0.7–0.8) for follicular; 0.5 (0.4–0.6) and 0.6 (0.4–0.8) for medullary; and 0.2 (0.1–0.3), 0.2 (0.2–0.3), and 0.2 (0.1–0.2) for anaplastic [18]. Mortality rates in Europe have remained consistently low and stable, and this trend can also be observed in several developed and developing countries [19].

Socioeconomic disparities in the incidence of TC arise because it is often discovered during routine screening or as an incidental finding in the assessment of other health conditions. Improved access to and utilization of health services increases the likelihood of overdiagnosis, leading to overdiagnosis and overtreatment. Globally, the overdiagnosis of TC is a problem that drives up the cost of medical care and is a major public health concern [17].

Therefore, new markers for TC need to be discovered to improve the detection of the disease at an early stage and to determine the diagnostic pathway and prognosis. This is essential for the early detection of the disease and offers the possibility of predicting disease progression and assessing the risk of aggressiveness. Metabolic disturbances are frequently observed in neoplasms, so the products of metabolism could serve as reliable markers for the disease. The crucial importance of lipidomics for diagnostic studies has been shown in various neoplasms. For example, the role of FAs in oncology has been evidenced in ovarian cancer. A growing number of studies demonstrate the link between lipid metabolism and an increased risk of PTC [20].

## 2. Reprogramming of Lipid Metabolism in TC

### 2.1. Fatty Acids

FAs are a fundamental component of almost all lipids, including triglycerides (TGs), phospholipids (PLs), sphingolipids (SPs), and esters of cholesterol [21]. In addition, they play an important role in cell structure and metabolic processes, as they are signaling molecules or substrates for energy metabolism. Since they are an active component of cell function, they are associated with carcinogenic signaling pathways [22]. Cancer cells often experience alterations in signaling pathways, which are frequently associated with changes in lipid metabolism. The reprogramming of FAs plays an important role in the mechanism underlying the development and progression of TC [23]. Most of the changes are associated with the number of polyunsaturated FAs (PUFAs). Researchers divide PUFAs into n-6 PUFA, which exerts a proinflammatory effect, and n-3 PUFA, which participates in metabolic pathways with anti-inflammatory properties [24]. Guo et al. observed lower levels of AA and adrenic acid (C22:4 n-6; AdA) in PTC and FTC tissues compared to adjacent normal thyroid tissue (Table 2) [25]. Moreover, the authors found higher levels of monounsaturated FAs (MUFAs), including oleic acid (C18:1 n-9), in cancer tissue compared to normal tissue [25]. Reduced levels of linoleic acid (C18:2 n-6, LA) and α-linolenic acid (C18:3 n-3, ALA) were found in the plasma of MTC [26]. LA inhibits tumor growth and formation by inducing apoptosis in tumor cells [27]. However, in MTC LA, a precursor of AA is reduced in the sera of patients with these cancers. AA is involved in the formation of prostaglandins, a class of oncogenic lipid signaling molecules [26]. Furthermore, the results of urinalysis obtained by Kim et al. confirm lower levels of LA and AA in TC patients compared to those of HCs and lower levels of AA and DHA in the urine of TC patients [28]. Changes in FA levels may indicate the malignancy of TC. Malignant lesions showed higher levels of saturated FAs (SFAs), including myristic acid (C14:0), C16:0, and ALA, but lower levels of dihomo-γ-linolenic acid (C20:3 n-6, DGLA) compared to benign thyroid lesions [29]. In addition, higher levels of C16:0 and stearic acid (C18:0) were observed in thyroid lesions compared to adjacent healthy tissues [29].

However, Wojakowska et al. showed that the tissue levels of SFAs, including C12:0, C14:0, pentadecanoic acid (C15:0), C16:0, heptadecanoic acid (C17:0), nonadecanoic acid (C19:0), and arachidic acid (C20:0), were downregulated in PTC, MTC, ATC, and FTC cancer patients compared to healthy tissue [32]. A similar result to Wojakowska et al. [32] with respect to C12:0 was shown by Xu et al. [30]. Higher levels of the abovementioned FAs were also observed in the sera of MTC patients compared to HC. Increased levels of SFA interfere with the cellular response induced by DNA damage in primary cells by causing the accumulation of p53 and reducing the induction of p21 and Bax, which enhances cell proliferation [21]. Changes in the levels of medium-chain FAs (MCFAs), including C10:0, were observed in the plasma of PTC patients compared to healthy plasma [37]. In addition, Abooshahab et al. indicated a correlation between capric acid (C10:0) and thyroid tumor progression [37]. Interestingly, the C18:1 level was lower in the cancerous tissues of PTC patients than in normal tissue [33], which is in contrast to the results of Guo et al. [25]. Similar observations regarding C16:1 were described by Tian et al. [31], who found a lower amount of C16:1 in contrast to Guo et al.’s results [25]. Lower concentrations of C16:1, C18:1, ALA, LA, C18:1, AA, and DHA were found in the sera of PTC patients compared to that of HCs [35].

### 2.2. Products of Lipid Oxidation

**Figure 2:**
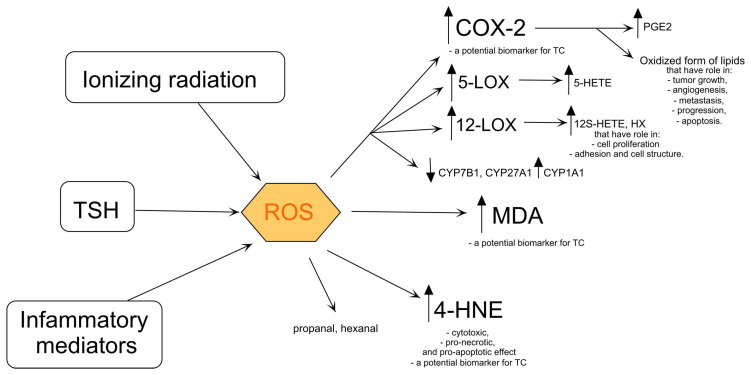
Products of lipid oxidation and its influence on cell metabolism. COX-2—cyclooxygenase-2, CYP1A1—aryl hydrocarbon hydroxylase, CYP7B1—25-hydroxycholesterol 7-alpha-hydroxylase, CYP27A1—sterol 27-hydroxylase, 5-HETE—5- hydroxyeicosatetraenoate, 12S-HETE—12S-hydroxyeicosatetraenoate, 4-HNE—4-hydroxy-2-nonenal, HX—hypoxylins, 5-LOX—5-lipooxygenase, 12-LOX—12-lipooxygenase, MDA—malondialdehyde, PGE2—prostaglandin E2, ROS—reactive oxygen species, TSH—thyroid-stimulating hormone. The arrows indicate that ionizing radiation, TSH, and inflammatory mediators lead to the production of ROS, which in turn activates COX-2 (producing PGE2), 5-LOX (producing 5-HETE), and 12-LOX (producing 12S-HETE and HX), while also increasing (↑) 4-HNE and MDA levels, and decreasing (↓) CYP7B1, CYP27A1, and increasing CYP1A1 activities.

Oxidative stress (OS) alters the composition of lipids, proteins, and nucleic acids, which can lead to the disturbance of homeostasis. Oxidized lipid products are formed as oxylipins in nonspecific reactions supported by OS and as a result of enzymatic reactions catalyzed by cyclooxygenases (COX), lipoxygenases (LOX), and cytochrome p450 (CYP450). The thyroid gland is an organ in which reactive oxygen species (ROS) are constantly formed under the influence of TSH (Figure 2). The increase in ROS levels determines the subsequent stages of the cancer [38,39]. Increased ROS levels, which are characteristic of PTC, are associated with aggressiveness. This could indicate the involvement of ROS in the development and progression of the disease [40,41]. ROS are actively involved in lipid peroxidation. The increase in lipid peroxidation may be due to the fact that the enzymes that perform peroxide sequestration cannot keep up with the high peroxide production [39]. Lipid peroxides are unstable compounds that rapidly decompose into 4-hydroxy-2-nonenal (4-HNE), malondialdehyde (MDA), propanal, and hexanal (Figure 2) [42]. Lipid peroxidation is higher in patients diagnosed with malignant tumors, including PTC [38,39], MTC [39], and FTC [39], than in the HC group. Elevated levels of MDA are observed in PTC and FTC [38,43] and PTC tissues [38,43] and in the sera of MTC patients [44]. PTC patients with metastases and angioinvasion are differentiated from patients with PTC without these features by the elevated MDA concentration [45]. The positive correlation between the MDA concentration and the lipid profile in the sera of PTC patients indicates the risk of a dysregulation of the lipid level, which in turn leads to the formation of metastases [46]. Furthermore, Stanley et al. observed an elevated MDA concentration in PTC patients unrelated to HT [39].

4-HNE is an important factor in carcinogenesis [47]. 4-HNE exhibits cytotoxic, pro-necrotic, and pro-apoptotic effects in tumors by regulating the activities of many genes, enzymes, and cytokines, involved in growth, regulation, and oxidative homeostasis [48,49]. Increased levels of 4-HNE were observed in TC tissues compared to healthy tissues [48]. Interestingly, Lopez et al. reported that high levels of 4-HNE were observed in PTC tissue compared to normal tissue [49]. PTC patients and PTC patients with HT had higher levels of 4-HNE in the thyroid gland than HCs. This may indicate that 4-HNE is a potential biomarker for differentiating malignant cancer from benign lesions (Figure 2) [49].

Oxidized forms of lipids generated by an enzymatic reaction catalyzed by COX-2 play an important role in tumor growth, angiogenesis, metastasis, progression, and apoptosis [50,51,52,53]. The overexpression of COX-2 is measured in the early phase of progression of any type of TC compared to normal thyroid tissue (Figure 2) [50] and nontoxic nodular goiter [54]. In addition, the expression of COX-2 is associated with malignant thyroid neoplasia in PTC, MTC, and FTC and is higher compared to healthy tissue [50,51,55] and in the Tpc-1 cell line (PTC) compared to the normal thyroid epithelial Nthy-ori-3-1 [56]. COX-2 is more highly expressed in PTC compared to FTC, ATC [51], benign thyroid tissue [57,58], and normal thyroid tissue [55]. The prognostic association of COX-2 expression was clearly pronounced in BRAF-mutated PTC compared to BRAF wild-type PTC [53]. COX-2 is involved in the formation of prostaglandin E2 (PGE2) (Figure 2). Sun et al. showed a higher concentration of PGE2 in PTC tumor tissue compared to adjacent non-tumor thyroid tissue [52]. The BRAF mutation could accelerate the formation of PGE2 with COX-2 [53]. Interestingly, another prostaglandin, prostaglandin I2 (PGI2), has anti-cancer properties [59]. Siironen et al. found that older patients with PTC (over 55 years) had significantly higher levels of COX-2 expression than younger patients who also had PTC (under 35 years) [60]. In comparison, Ito et al. showed lower COX-2 levels in older patients, in those with advanced stages of disease, and in patients with large tumors [51]. In addition, COX-2 expression was higher as the tumor progressed [60]. COX-2 may be a marker for TC, especially PTC (Figure 2) [61], and many authors have investigated its possible role in the carcinogenesis of PTC [56]. In vitro studies on TC cell lines showed that the selective COX-2 inhibitor, NS-398, can inhibit TC cell proliferation and migration as well as COX-2 expression in TC cells [62]. Moreover, further studies on PTC cell lines showed that NS-398 increased the expression of COX-2 mRNA and simultaneously inhibited tumor growth, indicating a role of COX-2 synthase and thromboxane A2 (TXA2) in the proliferation of PTC [62].

An increase in the expression of arachidonate 5-lipoxygenase (5-LOX) was observed in TC tissue compared to healthy tissue [63]. In addition, the overexpression of 5-LOX was observed in the tissues of PTC patients compared to adjacent normal tissue, indicating a role of 5-LOX in the progression of PTC [64]. Interestingly, the same studies showed that 5-LOX does not affect the growth and survival of TC cells [64]. On the other hand, a study by Kummer et al. suggests that 5-LOX may promote PTC progression through the 5-hydroxyeicosatetraenoate (5-HETE)-dependent (Figure 2) induction of matrix metalloproteinase-9 (MMP-9) [63]. 12-LOX is involved in the formation of 12S-hydroxyeicosatetraenoate (12S-HETE) and hypoxylins (HX) (Figure 2), including HXA3 and HXB3 [65]. The products of 12-LOX are involved in cell proliferation and adhesion and the maintenance of the cell structure and play a crucial role in cancer development and metastasis (Figure 2) [66]. The 12-LOX polymorphism may be a risk factor for TC [66]. The 12-LOX variant (AG variant) is more common in TC patients compared to HCs. This could indicate that the 12-LOX variant (AG) increases the risk of TC [66].

CYP450 is involved in the oxidative catalysis of the reactions of various endogenous and exogenous substances [67]. CYP450 catalyzes many reactions involved in the synthesis of cholesterol, steroids, and other lipids. One of the enzymes belonging to the CYP450 family is 25-hydroxycholesterol 7-alpha-hydroxylase (CYP7B1). In PTC, CYP7B1 was downregulated in aggressive tumor tissue, including PTC and ATC, compared to benign tumors, causing a high concentration of 27-hydroxycholesterol (Figure 2) [68]. The downregulation of CYP7B1 was also detected in the ATC cell line (CAL-62), and its overexpression reduced the growth and migration of CAL-62 [68]. Furthermore, the downregulation of sterol 27-hydroxylase (CYP27A1) completely blocked low-density lipoprotein-mediated cell proliferation [68]. Interestingly, the expression of aryl hydrocarbon hydroxylase (CYP1A1), the next enzyme in the CYP450 family, was increased in malignant tissue compared to benign tissue and was associated with metastasis and tumor size [67].

OS alters lipids, proteins, and nucleic acids, disrupting homeostasis. It leads to the formation of oxidized lipid products, including oxylipins, via COX, LOX, and CYP450 enzymes. The thyroid gland is constantly exposed to ROS due to TSH influence. The increased ROS levels are linked to cancer progression and aggressiveness in PTC. ROS involvement in lipid peroxidation results in unstable compounds like 4-HNE and MDA, which are elevated in PTC and correlate with metastasis and angioinvasion. COX-2 and 5-LOX enzymes, which are involved in prostaglandin and leukotriene production, play significant roles in tumor growth, angiogenesis, and metastasis. CYP450 enzymes also contribute to lipid metabolism alterations, influencing tumor development and progression.

### 2.3. Energy Sources

**Figure 3:**
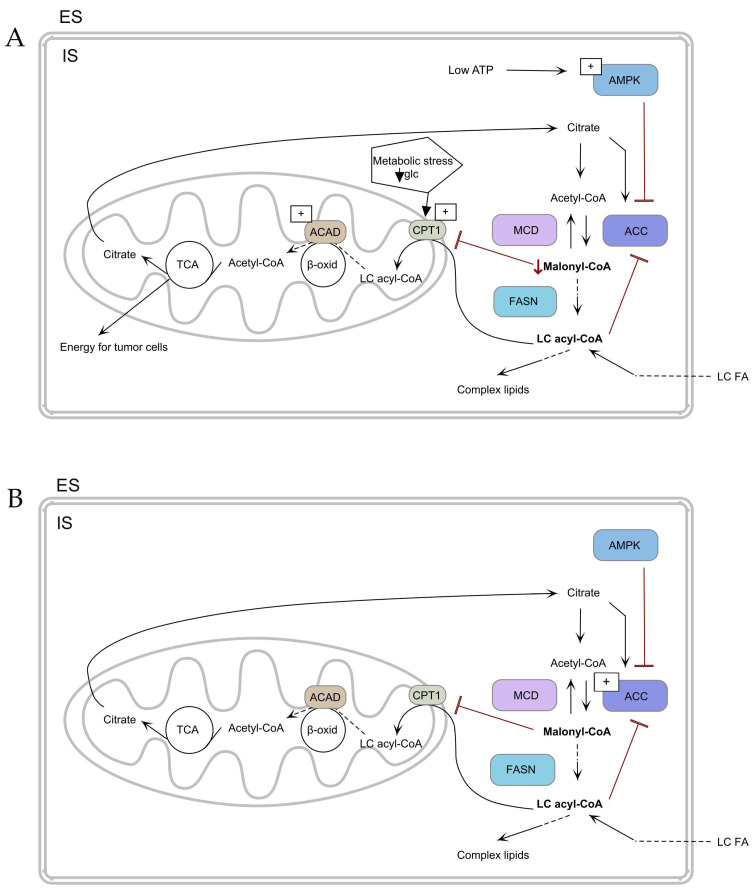
Mechanism of fatty acid uptake and metabolism (**A**) in TC cells and (**B**) in normal cells. ↓ Malonyl-CoA—decrease of Malonyl-CoA. TCA—tricarboxylic acid cycle, ACAD—acyl-CoA dehydrogenase, ACC—acetyl-CoA-carboxylase, AMPK—adenosine monophosphate–activated protein, β-oxid—β-oxidation, CPT1—carnitine palmitoyl transferase 1, ES—extracellular space, FASN—fatty acid synthase, glc—glucose, IS—intracellular space, MCD—malonyl-CoA decarboxylase, LC FA—long-chain fatty acid, LC acyl-CoA—long-chain acyl-CoA. The arrows depict the metabolic flux of fatty acids and related pathways. (**A**) Low ATP activates AMPK inhibiting ACC to reduce malonyl-CoA and glucose deprivation stimulate CPT1, shifting metabolism towards fatty acid oxidation for tumor cell energy. (**B**) ACC is activated, increasing malonyl-CoA levels and promoting lipid synthesis via FASN, with regulation by MCD.

In cancer tissue, energy metabolism is dysregulated, which is associated with a higher energy requirement for the rapid proliferation and development of cancer cells [69]. Cancer cells gain energy through the process of anaerobic glycolysis, the so-called Warburg effect, as well as in glutaminolysis [70]. The increase in anaplerosis indicates the mutual influence of glucose and glutamine metabolism [71]. The primary energy sources for the anaplerotic precursor of cancer are glutamic acid and aspartic acid, which are used in the TCA cycle. The TCA cycle provides energy for tumor cells (Figure 3A) [37]. The high glucose consumption and poor vascularization of the tumor result in limited nutrients and hypoxia, so other metabolic pathways must be used to ensure growth and survival. Glucose deficiency and metabolic stress promote both an increase in carnitine palmitoyl transferase 1 A (CPT1A) protein levels and an increase in CPT1C expression in the tumor tissues of patients with PTC compared to adjacent normal tissues (Figure 3B) [27,72]. However, Yao et al. showed that the amount of CPT1 was decreased in the sera of PTC patients compared to nodular goiter [36]. Interestingly, in the PTC cell line, including KTC-1, Cpt1c expression was also elevated compared to the vector from plasmid [73]. The increase in CPT1 will promote the β-oxidation process, which is correlated with the activation of the adenosine monophosphate–activated protein kinase (AMPK) metabolic pathway [73].

Zaugg Ket et al. indicated that AMPK activity under the conditions of hypoxia and glucose deprivation induces the expression of Cpt1c [74]. In addition, in ATC and PTC, AMPK inhibits ACC, which leads to a decrease in malonyl-CoA and consequently to the promotion of β-oxidation (Figure 3A) [75,76]. Another enzyme involved in energy metabolism is acyl-CoA dehydrogenases (ACADs). ACAD is one of the enzymes involved in β-oxidation [77]. Nagayama et al. found that the expression of ACAD was increased in PTC tissue compared to healthy tissue (Figure 3A,B) [78].

In cancer tissue, dysregulated energy metabolism meets heightened energy demands for the rapid proliferation and development of cancer cells. Anaplerosis, replenishing TCA cycle intermediates like glutamic acid and aspartic acid, supports tumor cell energy needs. Glucose scarcity and tumor hypoxia stimulate pathways like β-oxidation via increased CPT1 expression, which is crucial for ATP production and survival. AMPK activation under hypoxia and glucose deprivation enhances CPT1 expression and inhibits ACC, promoting β-oxidation in PTC and ATC. Elevated ACADs in PTC tissues further underscore altered energy metabolism in cancer cells.

### 2.4. Fatty Acid Uptake

This study has suggested that PTC cells have increased FA uptake compared to normal thyroid tissue. It is assumed that this increased uptake facilitates the heightened energy demands of rapidly proliferating cancer cells [79].

**Figure 4:**
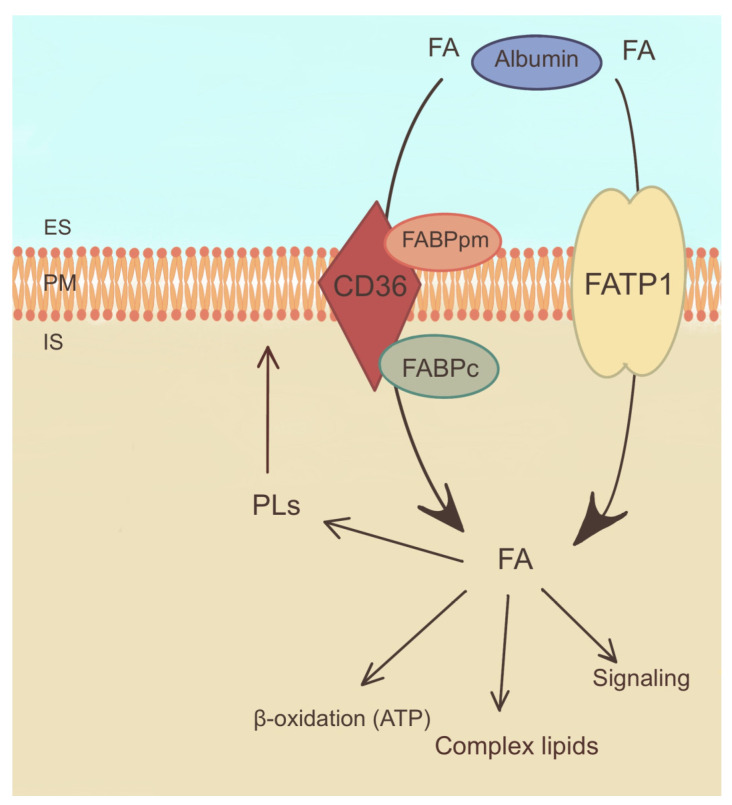
Mechanism of fatty acid uptake and metabolism. ES—extracellular space, FABPpm—plasma membrane fatty acid binding protein, FABPc—cellular fatty acid binding protein, FATP1—fatty acid transport protein 1, FA—fatty acid, PLs—phospholipids, PM—plasma membrane. The arrows indicate directions of fatty acid transport and their involvement in metabolic reactions.

The mechanisms underlying FA uptake in PTC involve several transporters and receptors that facilitate the entry of FAs into the cancer cells [80]. One of the key proteins involved in FA uptake is CD36, a transmembrane receptor that binds and internalizes FAs (Figure 4). The expression of CD36 has been found to be increased in PTC, possibly contributing to increased uptake of FAs [79]. Additionally, FA transport proteins (FATPs) have been associated with PTC as they facilitate the transport of FAs across cell membranes (Figure 4) [27].

Furthermore, FA binding proteins (FABPs) have been identified in PTC cells that support the intracellular transport and utilization of FA (Figure 4) [80]. These proteins help to shuttle acyl-CoA to various cellular compartments, including mitochondria, for energy production and storage [81].

The dysregulation of FA uptake in PTC has been linked to alterations in lipid metabolism and signaling pathways. It is hypothesized that an increased intake of FAs may contribute to the synthesis of membrane PLs, which are essential for the integrity and proliferation of cell membranes (Figure 4). Moreover, FAs can serve as substrates for the formation of bioactive lipid molecules, such as prostaglandins and leukotrienes, which are involved in inflammatory and tumorigenic processes [24].

AMPK is another important signal that activates catabolic processes in the cell under conditions of cellular metabolic stress. The activation of AMPK inhibits the proliferation of TC cells and, at the same time, facilitates their migration. Additionally, AMPK-regulated CPT1 facilitates the transfer of long-chain FAs into the mitochondria, promotes their oxidation, and improves the survival of TC cells under conditions of metabolic stress [79].

ATP-binding cassette subfamily A member 1 (ABCA1) is the main transporter responsible for cholesterol efflux from cells to high-density lipoprotein cholesterol (Figure 4). In aggressive PTC and ATC, ABCA1 is upregulated, suggesting its involvement in tumorigenesis [68]. ABCA1 plays a pivotal role in the regulation of cholesterol content and plasma membrane fluidity, which significantly influences the metastatic potential of cancer cells. Remarkably, while ABCA1 can promote metastasis through these mechanisms, it also exerts an inhibitory effect on tumor development by suppressing cell proliferation, highlighting the dual function of ABCA1 in regulating both proliferation and metastasis [82]. Further studies are essential to clarify the nuanced role of ABCA1 in the context of TC [68].

PTC cells have increased FA uptake compared to normal thyroid tissue, which supports the heightened energy needs of cancer cells. Key proteins involved in this process include CD36, FATPs, and FABPs, which facilitate FA entry and utilization within the cells. The dysregulation of FA uptake in PTC is linked to changes in lipid metabolism and signaling pathways, contributing to membrane synthesis and bioactive lipid production. AMPK and ABCA1 also play roles in FA metabolism and tumorigenesis, with AMPK promoting FA oxidation and ABCA1 regulating cholesterol efflux and influencing metastatic potential.

### 2.5. Polar Lipids

Polar lipids (PLs), including PLs and SPs, are a class of lipids that play a pivotal role in biological systems. They are major components of cell membranes and serve as structural building blocks, but they are also involved in various cellular processes [83]. The group of PLs includes phosphatidylcholines (PCs), phosphatidylethanolamines (PEs), phosphatidyl glycerols (PGs), phosphatidylserines (PSs), phosphatidylinositols (PIs), and phosphatidic acids (PAs) [84]. SPs include ceramide (Cer) and sphingomyelins (SMs) [85].

Lu et al. showed that the contents of PEs, PCs, PGs, PIs, SMs, and Cer in the tissue of PTC patients were comparable to those of the para-tumor tissues [27]. In the serum of PTC patients compared to HCs, the levels of PE (16:0/20:2), PE (O-18:0/18:3), PE (O-18:0/20:5), and PE (P-18:0/18:2) were downregulated, but PE-Nme (18:1/18:1) was upregulated (Table 3) [84]. According to Lee et al., PE (16:1p/22:6) was elevated in the sera of TC patients compared to that of HCs [86]. Interestingly, levels of PE (38:3), PE (38:4), and PE (40:6) were downregulated in the sera of PTC patients [86]. PEs are involved in the regulation of calcium transport in cell signaling, which tends to be restructured in TC cells [86]. Lysophosphatidylethanolamine (LPE) is a product of PE hydrolysis induced by phospholipase A1 (PLA1) or phospholipase A2 (PLA2) and is mainly a component of the cell membrane [87]. LPE (18:1) and LPE (18:2) were higher in the sera of TC patients [86].

Another phospholipid whose content changes in thyroid tumors is PC. PC plays a key role as a direct or indirect source of structural building blocks for the cell membrane and is responsible for maintaining the proliferation of tumor cells, thereby preserving the pro-tumoral properties that allow further tumor progression [88]. In malignant thyroid tissue from PTC and FTC, patients’ levels of PC (34:2), PC (36:2), PC (38:4), PC (38:6), PC (38:3), PC (34:1), and PC (32:0) were increased compared to adjacent non-tumor tissue [89]. Furthermore, the amount of PC (32:0), PC (32:1) [90], PC (34:1) [90,91], and PC (34:2) [91] was upregulated in tissues from PTC patients compared to adjacent normal tissues. Interestingly, PC levels allow for differentiation between FTC, PTC, benign tumors, and normal thyroid tissue [89,90,92]. In the sera of PTC and FTC patients, the levels of PC (34:2), PC (36:3), PC (34:1), PC (38:5), PC (36:1), PC (38:6), PC (35:2), and LPCs (20:4) were elevated compared to the sera of HCs [89]. The elevated levels of PC (34:1) and PC (36:1) may indicate that some lipids diffuse from the cancerous tissue into the blood [89]. Lower levels of PC (38:6) and PC (36:2) in malignant TC compared to normal tissue can be used as a biomarker for malignancy [89]. Interestingly, according to Lee et al., the serum level of LPC (18:2) was elevated compared to that of HCs [86]. Also, the content of PA changes in thyroid tissues. PA supports the regulation of intracellular membrane transport and controls changes in membrane fission and fusion. It can also be metabolized to DGA and lysophosphatidic acids, which have a major impact on membrane assembly [93]. In the tissues of PTC and FTC patients, the amounts of PA (38:3) [89], PA (36:2) [89,90], PA (36:3) [90], and PA (38:5) [89] were upregulated compared to the adjacent non-tumor tissue. PA (36:3) allows for differentiation between malignant cancer (PTC and FTC) and benign tumors [89,90,92]. Interestingly, in PTC and FTC patients’ sera, the levels of PA (36:3), PA (38:3), PA (38,4), PA (38:5), PA (40:5) PA (42:10), PA (40:6), and PA (36:2) were lower compared to the sera of HCs [89].

Some other PLs, such as PIs and lysophosphatidylinositols (LPIs), are able to activate signaling cascades relevant to cell proliferation, migration, survival, and tumorigenesis [87]. In TC serum, the levels of LPI (16:0), LPI (18:0), and LPI (18:1) were increased compared to healthy tissue, while the amount of PI (18:1/18:0) was downregulated [86]. In Lee et al., the level of PG (16:0/18:2) was shown to be increased in the plasma of PTC patients compared to the sera of healthy patients [86]. Another PL, SMs, are one of the biologically active signaling molecules involved in the regulation of cell growth, differentiation, or death. Accordingly, in a study by Jiang et al., the serum levels of SM (d18:1/15:0), SM (d18:1/16:1), and SM (d16:1/24:1) were elevated in patients with PTC compared to that of HCs [84]. An increased amount of SM (d18:1/22:0) [86] and a lower amount of SM (22:0) were found in the sera of patients with TC [89]. In the tissues of PTC patients, SM (34:1), SM (36:1) [90], and SM (d18:0/16:1) [91] were upregulated compared to healthy thyroid tissue. Interestingly, SMs (34:1) enabled the differentiation of PTC and FTC from benign thyroid tumors and normal tissue [89,90,92]. In conclusion, the total contents of PAs, PEs, LPEs, and SMs were increased, and PC content was lower in the plasma of PTC patients compared to that of HCs, which may indicate increased metabolism and the downregulation of β-oxidation [94]. Interestingly, PCs, PIs, PGs, PEs, and SMs were increased in tissues from PTC patients compared to para-tumor tissues [27].

SPs play a central role in cancer biology, including apoptosis, cell proliferation and migration [95]. Glycosphingolipids (GSPLs), including (Neu5Acα8Neu5Acα3Galβ4Glcβ1Cer) (GD3) ganglioside, have been found in MTC. GSPLs are higher in MTC compared to healthy thyroid tissue [96]. In addition, the Cer content was higher in the sera of PTC patients compared to that of HCs [94]. However, in PTC and FTC, Guo et al. showed that the level of CerP (d18:1/22:0) was decreased in patients’ sera compared to that of HCs [89]. Moreover, the level of Cer in the tissues of PTC patients was higher than in the tissues of para-tumors [27].

**Table 3:** Thyroid cancer-related changes in lipid species content in various biological samples.

Research Material	Fatty Acid	PTC		FTC		ATC		MTC	
		Direction of Change	Ref	Direction of Change	Ref	Direction of Change	Ref	Direction of Change	Ref
Tissues	PA (36:2)	↑	[25,89,90]	↑	[89]	nd		nd	
	PA (36:3)	↑	[90]	nd		nd		nd	
	PA(38:3)	↓	[25,89]	↓	[89]	nd		nd	
	PA(38:4)	↑	[89]	↑	[89]	nd		nd	
	PA(38:5)	↑	[89]	↑	[89]	nd		nd	
	PA(40:5)	↓	[25]	↓	[89]	nd		nd	
	PC(32:0)	↑	[89,90]	↑	[89]	nd		nd	
	PC(32:1)	↑	[25,90]	nd		nd		nd	
	PC(34:1)	↑	[25,89,90]	↑	[89]	nd		nd	
	PC(16:0/18:1)	↑	[91]	nd		nd		nd	
	PC(34:2)	↓	[89]	↓	[89]	nd		nd	
	PC(16:0/18:2)	↑	[91]	nd		nd		nd	
	PC(36:1)	↑	[90]	↑	[89]	nd		nd	
	PC(36:2)	↓	[89]	↓	[89]	nd		nd	
	PC(36:3)	↑	[90]	↓	[89]	nd		nd	
		↓	[89]						
	PC(38:3)	↓	[89]	↓	[89]	nd		nd	
	PC(38:4)	↓	[89]	↓	[89]	nd		nd	
	PC(38:6)	↑	[90]	↑	[89]	nd		nd	
	PE(38:4)	↓	[25]	nd		nd		nd	
	PE(42:5)	↓	[97]	nd		nd		nd	
	PI(38:4)	↓	[25]	nd		nd		nd	
	SM(22:0)	↓	[25,89]	↓	[89]	nd		nd	
	SM(24:1)	↓	[25,89]	↓	[89]	nd		nd	
	SM(34:1)	↑	[90]	nd		nd		nd	
	SM(d18:0/16:1)	↑	[91]	nd		nd		nd	
	SM(d18:1/16:0)	↑	[89]	↑	[89]	nd		nd	
	SM(36:1)	↑	[90]	nd		nd		nd	
	SM(d18:1/18:1)	↓	[89]	↓	[89]	nd		nd	
	MG(16:0)	↓	[32]	↓	[32]	↓	[32]	↓	[32]
	MG(18:0)	↓	[32]	↓	[32]	↓	[32]	↓	[32]
	4-HNE	↑	[49]	nd		nd		nd	
	MDA	↑	[38,43]	↑	[38,43]	nd		nd	
Serum	PA(36:2)	↓	[89]	↓	[89]	nd		nd	
	PA(36:3)	↓	[89]	↓	[89]	nd		nd	
	PA(38:3)	↓	[89]	↓	[89]	nd		nd	
	PA(38:4)	↓	[89]	↓	[89]	nd		nd	
	PA(38:5)	↓	[89]	↓	[89]	nd		nd	
	PA(40:5)	↓	[89]	↓	[89]	nd		nd	
	PA(42:10)	↓	[89]	↓	[89]	nd		nd	
	PC(32:0)	↑	[89]	↑	[89]	nd		nd	
	PC(34:1)	↑	[89]	↑	[89]	nd		nd	
	PC(34:2)	↑	[89]	↑	[89]	nd		nd	
	PC(35:2)	↑	[89]	↑	[89]	nd		nd	
	PC(36:1)	↑	[89]	↑	[89]	nd		nd	
	PC(36:3)	↑	[89]	↑	[89]	nd		nd	
	PC(38:5)	↓	[89]	↓	[89]	nd		nd	
	PC(38:6)	↓	[89]	↓	[89]	nd		nd	
	PC(40:6)	↓	[89]	↓	[89]	nd		nd	
	LPC(P-16:0)	↓	[36]	nd		nd		nd	
	LPC(16:0)	↓	[36]	nd		nd		nd	
	LPC(16:1)	↓	[36]	nd		nd		nd	
	LPC(18:0)	↓	[36]	nd		nd		nd	
	LPC(18:1)	↓	[36]	nd		nd		nd	
	LPC(18:3)	↓	[36]	nd		nd		nd	
	LPC(20:1)	↓	[36]	nd		nd		nd	
	LPC(20:4)	↓	[36]	nd		nd		nd	
	LPC(20:5)	↓	[36]	nd		nd		nd	
	LPC(22:5)	↓	[36]	nd		nd		nd	
	LPC(22:6)	↓	[36]	nd		nd		nd	
	SM(22:0)	↓	[89]	↓	[89]	nd		nd	
	CerP(d18:1/18:1)	↓	[89]	↓	[89]	nd		nd	
	MDA	nd		↑	[98]	nd		↑	[44]
Plasma	PC(O-14:0/15:0)	↓	[84]	nd		nd		nd	
	LPC(18:2)	nd	[86]	nd		nd		nd	
	PE(36:1)	↑	[86]	nd		nd		nd	
	PE(16:0/20:2)	↓	[84]	nd		nd		nd	
	PE(P-18:0/18:2)	↓	[84]	nd		nd		nd	
	PE(36:3)	↑	[86]	nd		nd		nd	
	PE(O- 18:0/18:3)	↓	[84]	nd		nd		nd	
	PE (38:3)	↓	[86]	nd		nd		nd	
	PE (38:4)	↓	[86]	nd		nd		nd	
	PE (18:0p/20:4)	↑	[86]	nd		nd		nd	
	PE(O-18:0/20:5)	↓	[84]	nd		nd		nd	
	PE (38:6)	↑	[86]	nd		nd		nd	
	PE(16:1p/22:6)	↑	[86]	nd		nd		nd	
	PE (40:6)	↓	[86]	nd		nd		nd	
	PE-NMe(18:1/18:1)	↑	[84]	nd		nd		nd	
	LPE(16:0)	nd	[86]	nd		nd		nd	
	LPE(18:1)	↑	[86]	nd		nd		nd	
	LPE(18:2)	↑	[86]	nd		nd		nd	
	PG(17:0/14:1)	↓	[84]	nd		nd		nd	
	PG(16:0/18:2)	nd	[86]	nd		nd		nd	
	PI(18:1/18:0)	↓	[84]	nd		nd		nd	
		nd	[86]						
	LPI(16:0)	nd	[86]	nd		nd		nd	
	LPI(18:0)	↑	[86]	nd		nd		nd	
	LPI(18:1)	↑	[86]	nd		nd		nd	
	PS(20:3/18:0)	↓	[84]	nd		nd		nd	
	PS(20:4/18:0)	↓	[84]	nd		nd		nd	
	SM(d18:1/15:0)	↑	[84]	nd		nd		nd	
	SM(d18:1/16:1)	↑	[84]	nd		nd		nd	
	SM(d18:1/20:0)	nd	[86]	nd		nd		nd	
	SM(d18:1/22:0)	↓	[86]	nd		nd		nd	
	SM(d16:1/24:1)	↑	[84]	nd		nd		nd	
	GlcCer(d14:1/24:1)	↑	[84]	nd		nd		nd	
	Sulfo HexCer(d18:1/22:0)	↓	[86]	nd		nd		nd	
	DG(12:1_18:0)	nd	[86]	nd		nd		nd	

4-HNE—4-hydroxy-2-nonenal, ATC—anaplastic thyroid cancer, DG—diacylglycerol, FTC—follicular thyroid cancer, LPC—lysophosphatidylcholine, LPE—lysophosphatidylethanolamine, LPI—lysophosphatidylinositol, MDA—malondialdehyde, MG—monoacylglycerol, MTC—medullary thyroid cancer, nd—no data, PA—phosphatic acid, PC—phosphatidylcholines, PE—phosphatidylethanolamine, PG—phosphatidyl glycerol, PI—phosphatidylinositol, PS—phosphoserine, PTC—papillary thyroid cancer, SM—sphingomyelin. Arrows indicate significant changes in lipid concentrations—whether increasing (↑) or decreasing (↓) —in serum, plasma or tissues.

### 2.6. Enzymes Involved in Lipid Metabolism

Enzymes are involved in various cellular processes, including cell growth, division, differentiation, and apoptosis (programmed cell death). The dysregulation of enzymes can disrupt the delicate balance of these processes and lead to uncontrolled cell growth and tumor formation [75].

**Figure 5:**
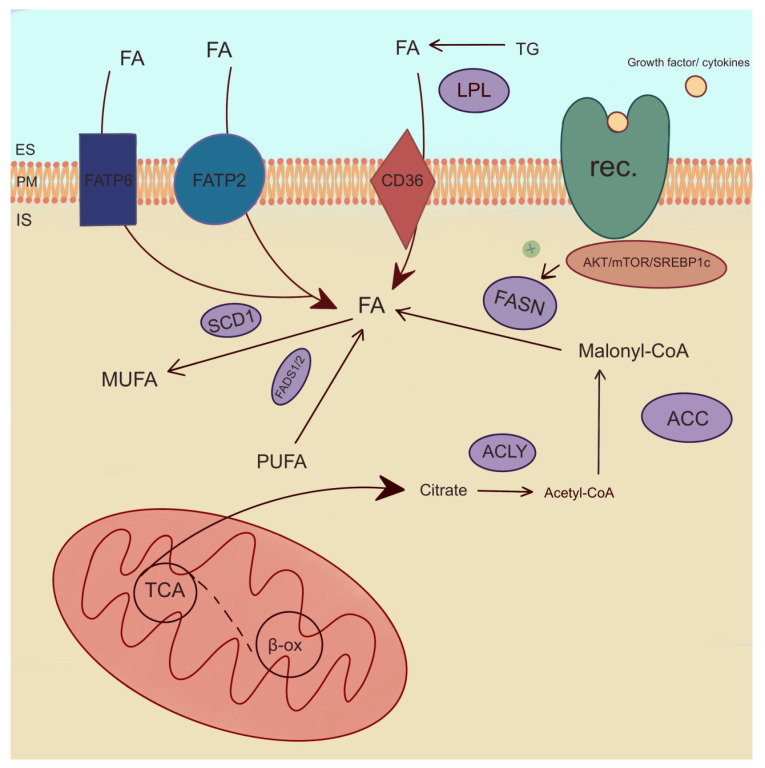
Enzymatic pathways of lipid metabolism. ACC—acetyl-CoA-carboxylase, ACLY—ATP-citrate lyase, β-ox—β-oxidation, IS—intracellular space, ES—extracellular space, FADS1/2—fatty acid desaturase 1 and 2, FASN—fatty acid synthase, FATP2,6—fatty acid transport protein 2,6, FA—fatty acid, LPL—lipoprotein lipase, MUFA—monounsaturated fatty acid, PM—plasma membrane, PUFA—polyusaturated fatty acid, rec.—receptor, SCD1—stearoyl-CoA desaturase 1, TCA—tricarboxylic acid cycle, TG—triglyceride. The arrows indicate directions of fatty acid transport and their involvement in metabolic reactions. The plus symbol denotes stimulation of FASN enzyme.

One of the enzymes involved in the de novo synthesis of FAs is acetyl-CoA-carboxylase (ACC). ACC is a rate-limiting enzyme for de novo lipid synthesis and the inhibition of β-oxidation (Figure 5) [99]. Studies have shown that a reduction in ACC2 levels in cells derived from PTCs by BRAFV600E leads to an increase in cancer cell proliferation [76]. The inhibition of BRAF increases ACC2 expression and lipid synthesis and decreases β-oxidation in cancer cell lines (BCPAP, KTC1, and TPC1) through potentially impaired cell proliferation [76]. FA synthase (FASN), as well as ACC, is involved in FA de novo formation. It catalyzes the synthesis of long-chain FAs, which are essential for cellular processes, including membrane formation and energy storage. FASN catalyzes the synthesis of palmitic acid and signal transduction via the AKT pathway (Figure 5) [100]. The gene-encoding FASN shows increased expression in various human cancers [25]. Interestingly, Liu et al. found that the knockdowns of PC reduce the level of FASN, as well as the level of FASN expression, by regulating the factor SREBP1c, which is activated in the AKT/mTOR/SREBP1c signaling pathway and is responsible for FA synthesis and TC progression (Figure 5) [101]. The overexpression of FASN indicates tumor progression in PTC, ATC, and FTC [100,102,103] and PTC cell line TPC1 [101], ONCO-DG-1, and B-CPAP [100], as well as ATC cell line 8505C [101] and ATC primers culture cell line collected from the thyroid tissue patient [102]. Interestingly, an increased expression of FASN and stearoyl-CoA desaturase 1 (SCD1) is associated with high levels of MUFA and monounsaturated PC in TC tissue compared to non-tumor tissue [25,89]. SCD1 catalyzes the process of the desaturation of SFAs into MUFAs (Figure 5). SCD1 plays a key role in the proliferation and viability that was researched in ATC cells, including THJ29T, THJ16T, and KTC2 [104]. In addition, SCD1 expression and SCD1 protein were increased in the tissue of ATC [104] and PTC [25,104] patients, as well as in several samples from FTC [104]. Ishikawa et al. showed that an increased level of SCD1 leads to an increase in C18:1 growth in pathogenic PTC tissue [91].

A consecutive important lipogenic enzyme is ATP-citrate lyase (ACLY), which plays a pivotal role in FA synthesis. ACLY is also critical for the stimulation of cell growth and cell aging [78]. The expression of ACLY is positively correlated with the cell proliferation pathway [105]. Interestingly, ACLY levels can be monitored as an indicator of cancer aggressiveness [106]. In PTC tissue, Huang et al. found no differences in the expression of ACLY compared to normal thyroid tissue. However, the upregulation of ACLY expression was observed in ATC [105]. One of the enzymes involved in FA transport is FA transport protein 2 (FATP2 or SLC27A2). FATP2 is overexpressed in TC, e.g., PTC, compared to healthy tissue [27,78,107]. In addition, FATP2 protein was increased in cancerous thyroid tissue. FATP2 is a poor prognostic factor for the progression and prognosis of thyroid tumors. This suggests that FATP2 captures and transports FA to PTC cells, thereby promoting tumor development [27]. In differentiated TC cell cultures, FATP2 also promoted proliferation and migration [107]. However, there were no significant differences in expression between PTC and FTC in malignant tissue [107]. FA transport protein 6 (FATP6 or SLC27A6) was also overexpressed in TC [107]. Moreover, FATP6 can be used as a potential biomarker for predicting the invasiveness of PTC [108].

Among lipogenic enzymes, the expression of lipoprotein lipase (LPL) was increased in PTC compared to healthy tissue [27,78]. LPL is an enzyme engaged in TG hydrolysis to FAs (Figure 5) [89]. Lu et al. observed a positive correlation between the increase in LPL expression and tumor growth and lymph node metastases by promoting hydrolysis TG [27]. This mechanism suggests that LPL is a poor prognostic factor for the development of PTC [27]. The FA binding protein (FABP4) is associated with poor overall survival [109]. In TC, the expression of FABP4 was lower compared to normal tissue [109]. Interestingly, FABP4 favors the development of TC by downregulating its suppressors, including BCL2, PTEN, and PPARG [110]. What is more, FABP4 promotes metastasis in thyroid malignancies [111,112]. In PTC, decreased FABP4 mediates the tumor-suppressive effect of PROX1 [113]. FABP4 may be a potential marker for TC [109].

FA desaturase 1 (FADS1) desaturates PUFAs n-3 and PUFAs n-6 at the delta-5 position, catalyzing the final step in the formation of EPA and AA (Figure 5) [114]. The expression of FADS1 was higher in TC tissue compared to that of HCs [115]. Interestingly, FA desaturase 2 (FADS2) catalyzed the first and rate-limiting step in the formation of tetracosapentaenoic acid (C24:5 n-3) and tetracosahexaenoic acid (C24:6 n-3) [114]. In thyroid lesions, FADS2 levels were upregulated compared to adjacent tissues [29]. PLA2 plays a role in PC metabolism [84]. It is more active in TC compared to normal thyroid tissue [84]. Interestingly, PLA2G2A and PL2G4A isoforms are associated with the pathogenesis of PTC, as they play a role in cell signaling and inflammatory response [116]. Ceramide synthase 2 (CerS2) is involved in sphingolipid metabolism and suppresses tumor metastasis [117]. The CerS2 contents in the tissues of PTC patients were higher compared to those of HCs. The studies conducted by Zeng et al. on TC cell lines indicate an inhibition of PTC cell proliferation and the occurrence of PTC metastases [117].

Metabolism in cancer cells is upregulated and requires substantial energy. FA, being an optimal energy source, is transported into cells via FATP2, FATP6, and CD36. Additionally, the enzymes catalyzing the conversion of FA, which are essential components of cell membranes and are engaged in cancer cell growth, are FADS1 and FADS2 causing PUFA desaturation by increasing the number of double bonds in the molecule, SCD1 catalyzing the conversion of SFA to MUFA and LPL hydrolyzing TG to glycerol and FA. Enzymes such as ACLY, FASN, ACC1, and ACC2 are involved in de novo FA synthesis. Moreover, FABP4 facilitates FA entry into cells and is implicated in the promotion of TC development. PLA2 plays a role in the inflammatory response, while ceramide synthase CerS2 is involved in sphingolipid metabolism, contributing to the suppression of tumor metastasis.

## 3. Effect of Hormones on TC Development and Lipid Metabolism

There is a close association between thyroid hormones and the development of TC. According to Huang et al., TSH was found to have a significant influence on PTC. The mean value of TSH concentration was higher in female PTC patients than in female HCs, while the amount of TSH was increased in male HCs compared to male PTC patients. The higher risk of PTC occurred in women with a TSH value below the normal range, while a higher risk was observed in men when the TSH value was above the normal range [118]. Several studies have found a link between sex hormones and the progression and development of TC. Estrogen receptors—ER-alfa and ER-beta—are overexpressed in TC cells. ER-alfa, which is activated by estrogen, is responsible for the proliferation, angiogenesis, and migration of cancer cells, while ER-beta, in contrast, has an antiproliferative effect. However, other sex hormones, such as progesterone and androgens, have no significant effect on the development and progression of TC. Further studies are needed [119].

## 4. FA Metabolism in TC in the Context of Obesity and Inflammation

Obesity and overweight are a growing global problem; as reported by the WHO in 2022, 2.5 billion adults were overweight, and 890 million people were classified as obese. The link between excessive fat accumulation and cancer is proven and well-known [120]. There are many studies that provide data on the relationship between TC and obesity, but only a minority explain the underlying molecular mechanism. Some of these include chronic inflammation, the secretion of adipokines and estrogens, the dysregulation of growth signaling pathways, altered immune response, and insulin and DNA damage from oxidative stress [121,122]. Furthermore, it has been shown that a higher body mass index (BMI) increases the risk of a larger primary tumor, microscopic extrathyroidal invasion, and advanced TNM staging [123]. There is a link between obesity and TC, and some authors mention possible factors, such as diabetes and/or insulin resistance, diet, cytokines, anthropometric components, and genetic variations, that could influence susceptibility to TC [121]. The role of adipose tissue is not limited to fat deposition, but it produces molecules involved in many metabolic processes. Most important in the context of cancer is its influence on inflammation. Adipose tissue is part of the immune system, as it expresses receptors for immune molecules [121]. Insulin-like growth factors (IGFs) are proteins with various physiological functions that are mainly involved in the regulation of growth and development. The IGF system is composed of several components, including two growth factors (IGF-1 and IGF-2), cell surface receptors (IGF-1R and IGF-2R), and six specific high-affinity binding proteins known as IGFBP-1 to IGFBP-6, as well as other IGF-binding molecules. The biological effects of IGF-1 are mediated via IGF-1R, a transmembrane protein that contains a tyrosine kinase domain. Upon activation, this domain initiates a series of events involving AKT, RAF-1/MEK/ERK proteins that form the major signaling pathway responsible for cancer proliferation and survival. Disruptions in the IGF axis are harbingers of tumor development in thyroid cells [122].

Patients with insulin resistance and hyperinsulinemia have an increased risk of many types of cancers, including TC. The underlying mechanism is probably that insulin serves not only as a metabolic hormone but also as a cell growth factor that can initiate the mitogenic cascade via activation of the MAPK and mTOR signaling pathways. This function is made possible by the binding of insulin to the following two receptors: IR-A, which recognizes both insulin and IGF 1 and 2, and IR-B, a receptor that is specifically responsible for insulin [122]. Leptin secreted by adipose tissue is similar in structure to other cytokines and is also involved in various metabolic processes, such as controlling food intake, stimulating angiogenesis, suppressing anti-inflammatory cytokines, and more. Leptin has been found to be associated with aggressive tumor pathological features and lymph node metastasis in DTC, as well as in PTC [124]. Resistin is another adipokine produced by human monocytes and macrophages that has proinflammatory, pro-proliferative, pro-angiogenic, and anti-apoptotic properties. The overexpression of this molecule therefore favors the development of neoplasms [125]. Adipose tissue mainly secretes adiponectin (APN), which is known for its anti-inflammatory properties and improving insulin sensitivity. Reduced APN plasma levels are associated with insulin resistance, which is observed in diseases such as obesity, diabetes, and atherosclerosis [126]. APN acts via the receptors AdipoR1 and AdipoR2, whereby AdipoR1 is widely distributed, and AdipoR2 is found more in hepatocytes and white adipose tissue [127]. Studies indicate that the positive role of APN on tumor tissues in endocrine cancers is through influencing intracellular cascades, inflammation, and insulin sensitivity. Lower APN levels are associated with obesity and increase the risk of TC. Prospective studies confirm the association between low APN and TC, especially in women. However, some studies on patients’ sera report no significant APN differences in benign nodules or PTC, although metabolic syndrome and higher BMI correlate with advanced stages of PTC [122].

## 5. Therapeutic Strategies Targeting Fatty Acid Metabolism

Although TC is characterized by a relatively low mortality rate, it presents a unique challenge due to its tendency to metastasize and affect adjacent organs. Exploring therapeutic strategies aimed at modifying FA metabolism in TC represents as a promising goal. Understanding the intricate metabolic pathways involved in FA metabolism may provide insight into potential interventions to prevent cancer progression and mitigate its impact on surrounding tissues. This brief introduction sets the stage for delving into the intricate landscape of therapeutic approaches targeting FA metabolism in TC.

### 5.1. Enzyme Inhibition

The inhibition of FASN emerges as a potential therapeutic approach as it suppresses PTC cell line C-75 proliferation and survival [100]. In addition to the de novo synthesis of lipids, cancer cells are dependent on the uptake of exogenous FA. CD36 as a FA transporter plays a crucial role in the uptake of FA into cancer cells, which promotes cell growth and proliferation. A similar role is also attributed to FABP and various lipoproteins. The inhibition of transporters reduces lipid uptake and impairs tumor cell growth [128].

Several studies have shown that the inhibition of ACC and acetyl-CoA synthetase is a promising method to delay disease progression and suppress tumorigenesis [27]. The use of a BRAFV300E inhibitor in PTC cells leads to the silencing of ACC2, which stops further cancer development [76].

In ATC, lovastatin inhibited 3-hydroxy-3-methylglutaryl-CoA reductase by inhibiting mevalonate synthesis and blocking geranylgeranylation and the subsequent activation of Rho-GTPase [75,129].

In addition, the inhibition of SCD1 led to a significant reduction in proliferation and induced cell death [104]. SCD1 inhibitors, including SI-4, betulinic acid, and MF-438, which have been shown to have anti-cancer effects, could be a promising therapy for the treatment of TC [130].

### 5.2. Dietetical Supplementation

As research progresses, there may be more evidence of the role of dietary supplementation and FA metabolism in PTC.

Omega-3 FAs, such as EPA and DHA, are PUFAs found in certain fish oils and marine sources. These FAs have been studied for their potential anti-inflammatory effects on cells by inhibiting cytokine production and NF-κB signaling in a human macrophage model. EPA and DHA reduced the production of proinflammatory cytokines associated with metabolic syndrome [131]. Metabolic syndrome is known to be associated with the development of neoplasms and the increased mortality of cancer [132].

The ketogenic diet is a high-fat, low-carbohydrate diet that aims to switch the body’s metabolism from using glucose as the primary energy source to using ketones derived from fats. The basic principle of the ketogenic diet in cancer treatment is based on the “Warburg effect”, which refers to the increased glycolysis (glucose metabolism) observed in cancer cells. By restricting carbohydrates and consuming large amounts of fats, the ketogenic diet aims to limit the availability of glucose, potentially starving the cancer cells and inhibiting their growth [133].

### 5.3. Hormones

One of the standard treatments for PTC is thyroid hormone suppression therapy. This involves administering synthetic thyroid hormones, such as L-T4 to suppress the production of TSH in the pituitary gland. By lowering the TSH level, the stimulation of the thyroid tissue, including the remaining cancer cells, is to be reduced and tumor growth inhibited [134]. The use of L-T4 has been shown to reduce cardiovascular risk and the incidence of TC in a group of patients aged 45–65 years [134]. Peroxisomal proliferator-activated receptor gamma (PPAR-γ) agonists, such as thiazolidinediones (TZDs), have been investigated in preclinical studies as potential therapeutics for PTC. TZDs are a group of drugs that increase insulin sensitivity by altering the hormone production of adipocytes, inducing the development of adipocytes and increasing adiponectin levels [135]. These drugs are ligands for the PPAR-γ receptor by inducing the upregulation of certain genes involved in the reduction of inflammation, insulin resistance, proliferation, and VEGF-induced angiogenesis in adipocytes. TZDs have been shown to inhibit PTC cell proliferation and induce cell death [135].

Since sex hormones influence the development of TC, they are also potential targets for targeted therapy. The use of aromatase inhibitors can address the significant impact of adipocyte aromatase activity in obesity on circulating estrogen, such as letrozole and anastrozole [119]. They have been used successfully in breast cancer, but their effects n TC are still unexplored and may prove to be an interesting target in the future [119].

## 6. Future Challenges

The role of dyslipidemia in the development of TC needs to be further explored and understood. Further research is needed to understand the exact background and molecular basis of the lipid metabolism that contributes to TC. Knowledge of the underlying processes of lipid metabolism may delay the progression of the disease and improve the efficacy of currently used methods, such as surgical conservative therapy or thermal ablation. Knowing the direction that diet and lifestyle should take in patients at high and very high risk of TC could prevent the development of the disease.

Targeted therapy is a promising alternative to conventional treatment, as it has a causal effect. It is important to know the potential biomarkers of TC, as they improve the detection of the disease at an early stage, the diagnostic pathway, and the prognosis.

These challenges require a solution to comprehensively address lipidomic disorders in thyroid neoplasms and introduce new, effective treatments.

## 7. Conclusions

Tumorigenesis in TC is associated with impaired FA metabolism, with lipogenic enzymes representing potential therapeutic targets. The inhibition of these enzymes can suppress the proliferation and progression of cancer cells. In addition, dietary supplementation with omega-3 FAs and the modulation of hormonal pathways, including thyroid hormone suppression therapy and PPAR-γ agonists, offer promising intervention options. Future challenges include further research into the molecular basis of lipid metabolism in TC and the development of targeted therapies. Understanding these metabolic disorders can improve early detection, diagnostic pathways, and prognosis. Overall, research into the complex interplay between FA metabolism, obesity, and inflammation has the potential to advance treatment strategies for TC.

## Figures and Tables

**Table 1:** Bethesda classification updated to align with the 2022 World Health Organization Classification of Thyroid Neoplasms [5].

Stage	Diagnostic Category
I	Nondiagnostic
II	Benign
III	Atypia of undetermined significance
IV	Follicular neoplasm
V	Suspicious for malignancy
VI	Malignant

**Table 2:** Thyroid cancer-related changes in fatty acid content in various biological samples.

Research Material	Fatty Acid	PTC		FTC		ATC		MTC		TC	
		Direction of Change	Ref	Direction of Change	Ref	Direction of Change	Ref	Direction of Change	Ref	Direction of Change	Ref
Tissues	C10:0	↓	[30]	nd		nd		nd		↑	[31]
	C12:0	↓	[30,32]	↓	[32]	↓	[32]	↓	[32]	nd	
	C14:0	↓	[32]	↓	[32]	↓	[32]	↓	[32]	↑	[31]
		↑	[23]								
	C15:0	↓	[32]	↓	[32]	↓	[32]	↓	[32]	nd	
	C16:0	↓	[32]	↓	[32]	↓	[32]	↓	[32]	↑	[31]
		↑	[23]								
	C17:0	↓	[32]	↓	[32]	↓	[32]	↓	[32]	nd	
	C18:0	nd		nd		nd		nd		↑	[31]
	C19:0	↓	[32]	↓	[32]	↓	[32]	↓	[32]	nd	
	C20:0	↓	[32]	↓	[32]	↓	[32]	↓	[32]	↑	[31]
	C22:0	nd		nd		nd		nd		↑	[31]
	C24:0	nd		nd		nd		nd		↑	[31]
	C16:1	↑	[25]	nd		nd		nd		↑	[31]
	C18:1	↑	[25]	nd		nd		nd		↑	[31]
		↓	[33]								
	C20:1	↑	[25]	nd		nd		nd		nd	
	C22:1	nd		nd		nd		nd		↑	[31]
	C24:1	nd		nd		nd		nd		↑	[31]
	C18:2 n-6	nd		nd		nd		nd		↑	[31]
	C20:2 n-6	↓	[30]	nd		nd		nd		nd	
	C20:3 n-6	↓	[23]	nd		nd		nd		nd	
	C20:4 n-6	↓	[25,34]	nd		nd		nd		↑	[31]
	C22:4 n-6	↓	[25]	nd		nd		nd		nd	
	C18:3 n-3	↑	[23,33]	nd		nd		nd		↑	[31]
	C20:5 n-3	nd		nd		nd		nd		↑	[31]
	C22:6 n-3	nd		nd		nd		nd		↑	[31]
	Ricinoleic acid	↓	[32]	↓	[32]	↓	[32]	↓	[32]	nd	
	alpha-aminoadipic acid	↑	[33]	nd		nd		nd		nd	
Serum	C16:1	nd		nd		nd		nd		↓	[35]
	C18:1	nd		nd		nd		nd		↓	[35]
	C20:1	↑	[36]	nd		nd		nd		nd	
	C18:2 n-6	nd		nd		nd		nd		↓	[35]
	C20:4 n-6	↓	[36]	nd		nd		nd		↓	[35]
	C18:3 n-3	↓	[36]	nd		nd		nd		↓	[35]
	C22:6 n-3	↓	[36]	nd		nd		nd		↓	[35]
	HVA	↓	[36]	nd		nd		nd		nd	
	3-HBA	↑	[36]	nd		nd		nd		nd	
Plasma	C16:0	nd		nd		nd		↓	[26]	nd	
	C18:0	nd		nd		nd		↓	[26]	nd	
	C18:2 n-6	nd		nd		nd		↓	[26]	nd	
	C20:4 n-6	nd		nd		nd		↓	[26]	nd	
	C18:3 n-3	nd		nd		nd		↓	[26]	nd	
Urinary	C18:2 n-6	nd		nd		nd		nd		↓	[28]
	C20:4 n-6	nd		nd		nd		nd		↓	[28]
	C18:3 n-3	nd		nd		nd		nd		↓	[28]
	C20:5 n-3	nd		nd		nd		nd		↓	[28]
	C22:6 n-3	nd		nd		nd		nd		↓	[28]

3-HBA—3-hydroxybutyric acid, ATC—anaplastic thyroid cancer, FTC—follicular thyroid cancer, HVA—hydroxyvaleric acid, MTC—medullary thyroid cancer, nd—no data, PTC—papillary thyroid cancer. Arrows indicate significant changes in fatty acid concentrations—whether increasing (↑) or decreasing (↓) —in serum, plasma or tissues.
